# Anterior Knee Pain Scale (AKPS): structural and criterion validity in Brazilian population with patellofemoral pain

**DOI:** 10.1186/s12891-024-07164-z

**Published:** 2024-01-08

**Authors:** Francisco Basilio da Silva-Júnior, Almir Vieira Dibai-Filho, Denise Carina Correa Barros, Jodimar Ribeiro dos Reis-Júnior, Matheus Bessa Smith Gonçalves, Alec Rodrigues Soares, Christian Emmanuel Torres Cabido, André Pontes-Silva, Cid André Fidelis-de-Paula-Gomes, Flavio de Oliveira Pires

**Affiliations:** 1 Postgraduate Program in Physical Education, Universidade Federal Do Maranhão, São Luís, Brazil; 2 Department of Physical Education, Universidade Federal Do Maranhão, São Luís, Brazil; 3 Academia The Club, São Luís, Brazil; 4 Postgraduate Program in Physical Therapy, Department of Physical Therapy, Universidade Federal De São Carlos, São Carlos, Brazil; 5 Postgraduate Program in Rehabilitation Sciences, Universidade Nove de Julho, São Paulo, Brazil

**Keywords:** Musculoskeletal disorders, Functional status, Factor analysis, statistical

## Abstract

**Purpose:**

To identify the best internal structure of the Brazilian version of the Anterior Knee Pain Scale (AKPS), comparing different instrument structures (structural validity) and correlating the scores of the versions (criterion validity).

**Methods:**

We included Brazilian volunteers, aged ≥ 18 years, with patellofemoral pain (PFP) for at least 3 months. We used the confirmatory factor analysis and considered the following fit indices: chi-square/degrees of freedom (DF), comparative fit index (CFI), Tucker-Lewis index (TLI), root mean square error of approximation (RMSEA). We considered the structure with the lowest values of the Akaike information criterion (AIC), sample size adjusted Bayesian information criterion (SABIC), and assessed criterion validity using Pearson correlation coefficient (r) to correlate the long and short versions.

**Results:**

The study included 101 participants, mostly women (65.3%), young adults (~ 31 years old), overweight (BMI > 25 kg/m^2^), incomplete higher education (37.6%), and physically active (64.4%). The original 1-domain, 13-item structure showed adequate fit indices (chi-square/GL < 3.00, TLI and CFI > 0.90, and RMSEA < 0, 08). However, items 11 and 12 had a factorial load of less than 0.23. Therefore, we excluded items 11 and 12 and found adequate fit indices (chi-square/GL < 3.00, TLI and CFI > 0.90, and RMSEA < 0, 08) and lower AIC and SABIC values. We observed a correlation coefficient above the acceptable cutoff of 0.70 (*r* = 0.966, *p*-value < 0.001) between the versions.

**Conclusion:**

The 11-item AKPS (without items 11 and 12) is the version with the most adequate internal structure and correlates satisfactorily with the long version of the instrument.

**Supplementary Information:**

The online version contains supplementary material available at 10.1186/s12891-024-07164-z.

## Introduction

Patellofemoral pain (PFP) is a common patellofemoral condition that is characterized by an insidious onset of poorly defined pain, localized to the anterior retropatellar and/or peripatellar region of the knee [1]. The onset of symptoms may be slow or acutely develop with a worsening of pain accompanying lower-limb loading activities [2].

The Anterior Knee Pain Scale (AKPS), or Kujala score, was developed by Kujala et al. [3] in Finland (in English, in 1993) to measure aspects of disability [4] caused by patellofemoral disorders (e.g., PFP, patellar subluxation, and patellar dislocation). The authors validated the construct by finding significantly lower scores in patients with patellofemoral disorders when compared to a control group.

The original instrument has 13 items and scores ranging from 0 to 100, with lower scores indicating greater disability. Since the development of the instrument [3], several studies have been conducted to adapt the AKPS to other cultures, including Turkish [5], Chinese [6], Persian [7], Spanish [8], Dutch [9], Thai [10], Greek [11], Arabic [12], Indonesian [13], Norwegian [14], Italian [15], German [16], and French [17].

In Brazil, two studies translated and adapted the AKPS for the population with PFP. Firstly, Aquino et al. [18] performed the translation and cross-cultural adaptation and did not investigate any other measurement properties. Subsequently, a more robust study translated and adapted the AKPS for the Brazilian population and identified the instrument with adequate internal consistency and reliability, with a valid construct, and with satisfactory responsiveness [4]. However, no validation studies have examined the internal structure of the AKPS through factor analysis, thus reducing its relevance to clinical or research settings.

A systematic review examined the measurement properties of scales and questionnaires for patients with PFP and found that only the Activities of Daily Living Scale (ADLS) and the International Knee Documentation Committee (IKDC) had an internal structure confirmed by factor analysis [19]. Furthermore, according to Hoglund et al. [1, 20], most patient-reported outcome measures used to measure pain and function in patients with PFP have inadequate content validity.

The evaluation of condition-specific patient-reported outcomes is highly recommended by experts [21], with the AKPS being one of the most commonly used instruments and correlating with many physical and non-physical factors [22, 23]. However, the assessment of the measurement properties of AKPS and other patient-reported outcome measures for knee disorders is scarce, as shown by systematic reviews [1, 19, 20].

Therefore, considering this gap in the literature, the objective of the present study was to identify the best internal structure of the Brazilian version of the AKPS, comparing different instrument structures (structural validity) and correlating the scores of the versions (criterion validity) whose measurement provides an adequate indication of the dimensionality of the construct, attribute or factor being measured.

## Methods

### Study design and ethical aspects

A cross-sectional study to examine the structural validity of the AKPS. Data collection for the study was done using an online form (Google Forms, Mountain View, CA, USA)®. Participants were recruited through advertising in the university community, rehabilitation clinics, and gyms in São Luís (Maranhão, Northeastern Brazil). Social media advertising was also used. The study procedures were approved by the Institutional Research Ethics Committee (protocol number 3.995.226).

### Sample size

The sampling was based on the recommendations of the COnsensus-based Standards for the selection of health Measurement INnstruments (COSMIN) [19]. Namely, seven times the number of items of the scale, provided that this value is not less than 100. In this sense, considering 13 items, the present study was composed of at least 100 individuals with PFP.

### Eligibility criteria

We included only participants with PFP [2] and adopted the following exclusion criteria: history of trauma, fracture, or acute injury to the knee joint; knee surgery; use of analgesics in the past seven days; physiotherapy treatment for PFP in the past three months; and presence of other chronic pain. The diagnosis was made asynchronously and remotely.

### Assessments

Participants reported whether they engaged in physical activity (yes or no), the number of times per week, the duration, and the type of exercise. Afterward, a bachelor of physical education (AP-S) evaluated the reports and classified them according to relevant recommendations for physical activity levels [24].

In addition to the Numerical Pain Rating Scale (NPRS), which assesses the mean pain intensity of the participants, and an initial assessment of personal, sociodemographic, anthropometric and clinical aspects (Table 2), we used the AKPS, a scale adapted to Brazilian Portuguese [4] with 13 items and different response possibilities for each item, corresponding to a specific score, as shown in Table 1. The final score of the scale is obtained by adding the score of each item, ranging from 0 to 100. Lower scores indicate greater disability.

**Table 1:** Score of responses for each item on the Anterior Knee Pain Scale (AKPS)

Item	Score
1. Limp	a) 5 b) 3 c) 0
2. Support	a) 5 b) 3 c) 0
3. Walking	a) 5 b) 3 c) 2 d) 0
4. Stairs	a) 10 b) 8 c) 5 d) 0
5. Squatting	a) 5 b) 4 c) 3 d) 2 e) 0
6. Running	a) 10 b) 8 c) 6 d) 3 e) 0
7. Jumping	a) 10 b) 7 c) 2 d) 0
8. Prolonged sitting with the knees flexed	a) 10 b) 8 c) 6 d) 4 e) 0
9. Pain	a) 10 b) 8 c) 6 d) 3 e) 0
10. Swelling	a) 10 b) 8 c) 6 d) 4 e) 0
11. Abnormal painful kneecap (patellar) movements (subluxations)^a^	a) 10 b) 6 c) 4 d) 2 e) 0
12. Atrophy of thigh^a^	a) 5 b) 3 c) 0
13. Flexion deficiency	a) 5 b) 3 c) 0

^a^Excluded items

Therefore, participants of both sexes, sedentary or active, aged between 18 and 60 years, and with reports of PFP [2] for at least 3 months were included. In addition to the participant's verbal report, the NPRS was used to characterize the participant’s pain intensity: a unidimensional scale from 0 to 10 points, where 0 represents "no pain" and 10 represents "worst pain imaginable", with adequate validity for the Portuguese population [25].

### Statistical analysis

We performed descriptive analysis and presented data as means and standard deviations or relative and absolute frequencies. We used confirmatory factor analysis (CFA) to identify the best structure of the AKPS through R Studio software (Boston, MA, USA)®, using the lavaan and semPlot packages. We used the implementation of a polychoric matrix and the robust diagonally weighted least squares (RDWLS) extraction method [26, 27]. We considered appropriate values of fit indices for the following cut-offs: chi-square/degrees of freedom (DF) < 3; comparative fit index (CFI) and Tucker-Lewis index (TLI) > 0.90; and root mean square error of approximation (RMSEA) < 0.08 [28, 29].

For model comparison, the structure with the lowest Akaike Information Criterion (AIC) and Sample Size Adjusted Bayesian Information Criterion (SABIC) values was considered most appropriate [30]. Factor loadings were considered adequate if they were greater than 0.40 [31]. Finally, we assessed validity criteria using the 13-item long version of the AKPS as the gold standard. Therefore, we used the Pearson correlation coefficient (r) to correlate the long and short versions (data with normal distribution according to the Kolmogorov–Smirnov test). A correlation coefficient > 0.70 was considered an appropriate cut-off point for criterion validity [32].

## Results

The AKPS proposed in this study consists of 11 items with separate categories related to different levels of knee function. Categories within each item are scored and responses are summed to produce a global index where a score of 85 represents "no deficit" and a score of 0 represents "the highest possible deficit". Response scores for each item on the AKPS are shown in Table 1.

The majority of the sample consists of women (65.3%), young adults (~ 31 years old), overweight (body mass index > 25 kg/m2), with incomplete higher education (37.6%), and practitioners of physical activity (64.4%) (Table 2). Reading the pain characteristics (Table 3), we observed that the majority of the sample has pain in the sitting position (82.2%) or crouching (67.3%), mean pain duration greater than 39 months, and mean pain intensity greater than 4 points on the NPRS. Regarding the side of greater pain, there was a similar distribution between unilateral pain on the right (32.7%), on the left (36.6%), and bilateral (30.7%).

**Table 2:** Personal and anthropometric characteristics of the sample (*n* = 101)

Variable	Mean (standard deviation) or n (%)
Age (years)	31.77 (12.21)
Body mass (kg)	71.07 (15.19)
Stature (m)	1.65 (0.08)
Body mass index (kg/m^2^)	25.75 (4.39)
Sex	
Male	35 (34.7%)
Female	66 (65.3%)
Education	
Incomplete primary education	1 (1%)
Complete primary education	1 (1%)
Incomplete secondary education	1 (1%)
Complete secondary education	12 (11.9%)
Incomplete higher education	38 (37.6%)
Complete higher education	26 (25.7%)
Incomplete post-graduate	5 (5%)
Complete post-graduate	17 (16.8%)
Lower limb dominance	
Right	79 (78.2%)
Left	13 (12.9%)
Both	9 (8.9%)
Physical activity	
Yes	65 (64.4%)
No	36 (35.6%)

**Table 3:** Sample pain characteristics

Variables	Mean (standard deviation) or n (%)
Pain presence	
Sitting (yes)	83 (82.2%)
Crouched (yes)	68 (67.3%)
Running (yes)	62 (61.4%)
Jumping (yes)	62 (61.4%)
Up or down stairs (yes)	66 (65.3%)
Time of pain (months)	39.04 (50.38)
Knee in pain	
Right	33 (32.7%)
Left	37 (36.6%)
Both	31 (30.7%)
AKPS	
13 items (score, 0–100)	76.00 (13.09)
11 items (score, 0–85)	63.74 (12.21)
NPRS at rest (score, 0–10)	4.51 (1.99)

*AKPS* Anterior Knee Pain Scale, *NPRS* Numerical Pain Rating Scale

**Fig. 1:**
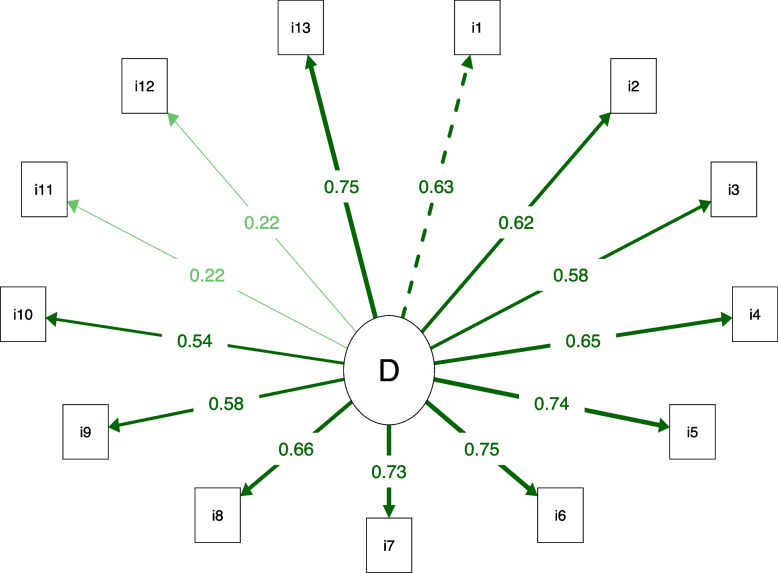
Path diagram of the Anterior Knee Pain Scale (AKPS) with 13 items. All factor loadings above 0.40, except items 11 and 12. The dotted line indicates the first factor item. The thicker the line, the greater the factor loading. D: Disability

**Fig. 2:**
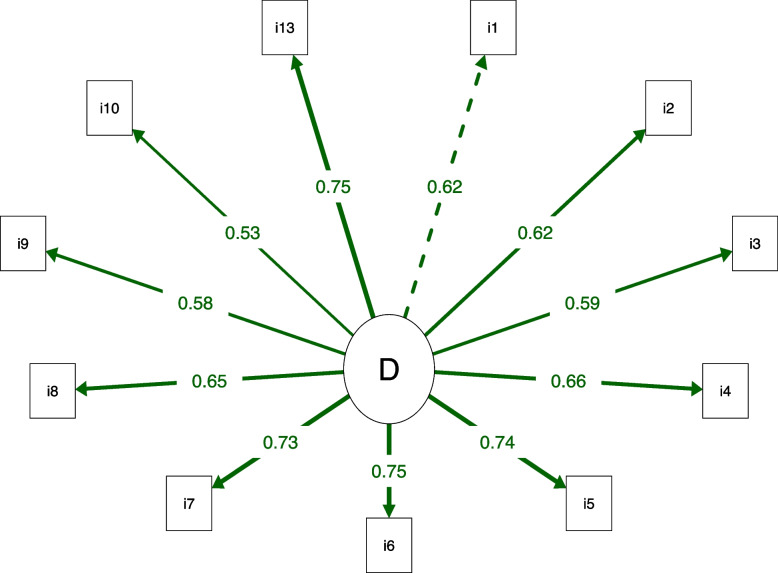
Path diagram of the Anterior Knee Pain Scale (AKPS) with 11 items. All factor loadings above 0.40. The dotted line indicates the first factor item. The thicker the line, the greater the factor loading. D: Disability

Regarding the internal structure of the AKPS (Table 4), the structure with 1 domain and 13 items showed adequate fit indices (Chi-square/GL < 3.00, TLI and CFI > 0.90, and RMSEA < 0.08). However, items 11 and 12 had a factorial load of less than 0.23, indicating that they were poorly explained by the domain (Fig. 1). Therefore, we excluded items 11 and 12 and found adequate fit indices (chi-square/GL < 3.00, TLI and CFI > 0.90, and RMSEA < 0.08) and lower AIC and SABIC values (Table 4), in addition to factor loadings greater than 0.40 (Fig. 2). Thus, the AKPS structure with 1 domain and 11 items is more appropriate (Additional file 1).

**Table 4:** Confirmatory factor analysis of the versions of the Anterior Knee Pain Scale (AKPS)

Structure	Chi-square/DF	CFI	TLI	RMSEA (90% CI)	AIC	SABIC
13 items	1.35	0.965	0.958	0.059 (0.019 to 0.089)	4824.880	4810.754
11 items	1.45	0.970	0.962	0.067 (0.024 to 0.101)	4043.385	4031.432

*DF* Degree of freedom, *CFI* Comparative fit index, *TLI* Tucker-Lewis index, *RMSEA* Root mean square error of approximation, *CI* Confidence interval, *AIC* Akaike information criterion, *SABIC* Sample-size adjusted Bayesian information criterion

In terms of criterion validity, the correlation between the 11-item and 13-item versions of the AKPS showed a correlation coefficient above the acceptability cutoff of 0.70 (*r* = 0.966, *p*-value < 0.001). Thus, even with the reduction of two items, the final scores remain highly correlated.

## Discussion

This study showed that the 11-item AKPS, excluding items 11 and 12, is the version with the most adequate internal structure and satisfactorily correlated with the long version of the instrument. Although AKPS has been adapted for Turkish [5], Chinese [6], Persian [7], Spanish [8], Dutch [9], Thai [10], Greek [11], Arabic [12], Indonesian [13], Norwegian [14], Italian [15], German [16], and French [17], the authors did not analyze the internal structure of the AKPS. To date, this is the first validation study to examine the internal structure of the AKPS using factor analysis.

We emphasize that the clinical and scientific relevance of the AKPS is still limited because important measurement properties (e.g., reproducibility, responsiveness, and interpretability) of the AKPS still need to be investigated [1, 20]. Therefore, we suggest that further studies are needed to determine whether this instrument should be added to or removed from the scientific community.

This study has important limitations. First, according to the American College of Rheumatology [33], clinical symptoms related to degenerative lesions in the knee joint begin to appear at 38 years of age, with radiographic evidence becoming detectable at 40 years of age or older; thus, generalizability to the PFP population, which consists primarily of young individuals [34], may be compromised.

Second, although the results support the 11-item AKPS as the version with the most adequate internal structure and satisfactory correlation with the long version of the instrument, we know that the 11-item AKPS has adequate measurement properties only for the Brazilian population. Since this is the first validation study to examine the internal structure of the AKPS through factor analysis, we propose its reproducibility in other countries and its comparison with other instruments. Finally, the sample was predominantly female, and future studies should balance the number of participants by gender.

## Conclusion

The 11-item AKPS (without items 11 and 12) is the version with the most adequate internal structure and correlates satisfactorily with the long version of the instrument.

### Supplementary Information

**Additional file 1.** The 11-item Anterior Knee Pain Scale (AKPS). Anterior Knee Pain Scale (AKPS) com 11 items.

## Data Availability

The data and materials in this paper are available from the corresponding author on request.

## Abbreviations

AIC: Akaike Information Criterion
AKPS: Anterior Knee Pain Scale
SABIC: Sample-Size Adjusted Bayesian Information Criterion
CFA: Confirmatory Factor Analysis
CFI: Comparative Fit Index
COSMIN: COnsensus-based Standards for the selection of health Measurement INstruments
DF: Degree of freedom
NPRS: Numeric Pain Rating Scale
PFP: Patellofemoral pain
RDWLS: Robust Diagonally Weighted Least Squares
R: Pearson correlation coefficient
RMSEA: Root Mean Square Error of Approximation
TLI: Tucker-Lewis index

## References

[1] Hoglund LT, Scalzitti DA, Jayaseelan DJ, Bolgla LA, Wainwright SF (2023). Patient-reported outcome measures for adults and adolescents with patellofemoral pain: a systematic review of construct validity, reliability, responsiveness, and interpretability using the COSMIN methodology. J Orthop Sports Phys Ther.

[2] Willy RW, Hoglund LT, Barton CJ, Bolgla LA, Scalzitti DA, Logerstedt DS (2019). Patellofemoral pain. J Orthop Sports Phys Ther..

[3] Kujala UM, Jaakkola LH, Koskinen SK, Taimela S, Hurme M, Nelimarkka O (1993). Scoring of patellofemoral disorders. Arthroscopy.

[4] Da Cunha RA, Pena Costa LO, Hespanhol Junior LC, Pires RS, Kujala UM, Lopes AD (2013). Translation, cross-cultural adaptation, and clinimetric testing of instruments used to assess patients with patellofemoral pain syndrome in the Brazilian population. J Orthop Sports Phys Ther.

[5] Kuru T, Dereli EE, Yaliman A (2010). Validity of the Turkish version of the Kujala patellofemoral score in patellofemoral pain syndrome. Acta Orthop Traumatol Turc.

[6] Cheung RTH, Ngai SPC, Lam PL, Chiu JKW, Fung EYH (2012). Chinese translation and validation of the Kujala scale for patients with patellofemoral pain. Disabil Rehabil.

[7] Negahban H, Pouretezad M, Yazdi MJS, Sohani SM, Mazaheri M, Salavati M (2012). Persian translation and validation of the Kujala patellofemoral scale in patients with patellofemoral pain syndrome. Disabil Rehabil.

[8] Gil-Gámez J, Pecos-Martín D, Kujala UM, Martínez-Merinero P, Montañez-Aguilera FJ, Romero-Franco N (2016). Validation and cultural adaptation of “Kujala score” in Spanish. Knee Surg Sports Traumatol Arthrosc.

[9] Ummels PEJ, Lenssen AF, Barendrecht M, Beurskens AJHM. Reliability of the Dutch translation of the Kujala patellofemoral score questionnaire. Physiother Res Int. 2017;22:1. 10.1002/pri.1649 26308151

[10] Apivatgaroon A, Angthong C, Sanguanjit P, Chernchujit B (2016). The validity and reliability of the Thai version of the Kujala score for patients with patellofemoral pain syndrome. Disabil Rehabil.

[11] Papadopoulos C, Constantinou A, Cheimonidou AZ, Stasinopoulos D (2017). Greek cultural adaption and validation of the Kujala anterior knee pain scale in patients with patellofemoral pain syndrome. Disabil Rehabil.

[12] Alshehri A, Lohman E, Daher NS, Bahijri K, Alghamdi A, Altorairi N (2017). Cross-cultural adaptation and psychometric properties testing of the Arabic anterior knee pain scale. Med Sci Monit.

[13] Mustamsir E, Phatama KY, Pratianto A, Pradana AS, Sukmajaya WP, Pandiangan RAH (2020). Validity and reliability of the Indonesian version of the Kujala score for patients with patellofemoral pain syndrome. Orthop J Sports Med.

[14] Hott A, Liavaag S, Juel NG, Brox JI, Ekeberg OM (2021). The reliability, validity, interpretability, and responsiveness of the Norwegian version of the Anterior Knee Pain Scale in patellofemoral pain. Disabil Rehabil.

[15] Cerciello S, Corona K, Morris BJ, Visonà E, Maccauro G, Maffulli N (2018). Cross-cultural adaptation and validation of the Italian versions of the Kujala, Larsen, Lysholm and Fulkerson scores in patients with patellofemoral disorders. J Orthop Traumatol..

[16] Dammerer D, Liebensteiner MC, Kujala UM, Emmanuel K, Kopf S, Dirisamer F (2018). Validation of the German version of the Kujala score in patients with patellofemoral instability: a prospective multi-Centre study. Arch Orthop Trauma Surg.

[17] Buckinx F, Bornheim S, Remy G, Van Beveren J, Reginster J, Bruyère O (2019). French translation and validation of the “Anterior Knee Pain Scale” (AKPS). Disabil Rehabil.

[18] Aquino V da S, Falcon SFM, Neves LMT, Rodrigues RC, Sendín FA (2011). Tradução e adaptação cultural para a língua portuguesa do questionário scoring of patellofemoral disorders: estudo preliminar. Acta Ortop Bras..

[19] Esculier JF, Roy JS, Bouyer LJ (2013). Psychometric evidence of self-reported questionnaires for patellofemoral pain syndrome: a systematic review. Disabil Rehabil.

[20] Hoglund LT, Scalzitti DA, Bolgla LA, Jayaseelan DJ, Wainwright SF (2023). Patient-reported outcome measures for adults and adolescents with patellofemoral pain: a systematic review of content validity and feasibility using the COSMIN methodology. J Orthop Sports Phys Ther.

[21] Barton CJ, De Oliveira SD, Morton S, Collins NJ, Rathleff MS, Vicenzino B (2021). REPORT-PFP: a consensus from the international patellofemoral research network to improve REPORTing of quantitative PatelloFemoral pain studies. Br J Sports Med.

[22] Botta AFB, de Cássia Pinto da Silva J, Dos Santos Lopes H, Boling MC, Briani RV, de Azevedo FM (2023). Group-and sex-related differences in psychological and pain processing factors in people with and without patellofemoral pain: correlation with clinical outcomes. BMC Musculoskelet Disord..

[23] Nakagawa TH, Serrão FV, Maciel CD, Powers CM (2013). Hip and knee kinematics are associated with pain and self-reported functional status in males and females with patellofemoral pain. Int J Sports Med.

[24] Maciel E da S, Silva BKR, Figueiredo FWDS, Pontes-Silva A, Quaresma FRP, Adami F (2022). Physical inactivity level and lipid profile in traditional communities in the Legal Amazon: a cross-sectional study : Physical inactivity level in the Legal Amazon. BMC Public Health..

[25] Ferreira-Valente MA, Pais-Ribeiro JL, Jensen MP (2011). Validity of four pain intensity rating scales. Pain.

[26] Li CH (2016). Confirmatory factor analysis with ordinal data: comparing robust maximum likelihood and diagonally weighted least squares. Behav Res Methods.

[27] Ullman J (2006). Structural equation modeling: reviewing the basics and moving forward. J Pers Assess.

[28] Brown T (2006). Confirmatory factor analysis for applied research.

[29] Schermelleh-Engel K, Moosbrugger H, Müller H (2003). Evaluating the fit of structural equation models: Tests of significance and descriptive goodness-of-fit measures. MPR-Online..

[30] Araujo GGC, Fidelis-de-Paula-Gomes CA, Pontes-Silva A, Pinheiro JS, Mendes LP, Gonçalves MC (2021). Brazilian version of the neck Bournemouth questionnaire does not have a well-defined internal structure in patients with chronic neck pain. Clin Rehabil..

[31] Barreto FS, Avila MA, Pinheiro JS, Almeida MQG, Ferreira C de SB, Fidelis-de-Paula-Gomes CA (2021). Less is more: five-item neck disability index to assess chronic neck pain patients in Brazil. Spine (Phila Pa 1976)..

[32] Prinsen CAC, Mokkink LB, Bouter LM, Alonso J, Patrick DL, de Vet HCW (2018). COSMIN guideline for systematic reviews of patient-reported outcome measures. Qual Life Res.

[33] Bijlsma JWJ, Berenbaum F, Lafeber FPJG (2011). Osteoarthritis: an update with relevance for clinical practice. Lancet.

[34] Smith BE, Selfe J, Thacker D, Hendrick P, Bateman M, Moffatt F (2018). Incidence and prevalence of patellofemoral pain: a systematic review and meta-analysis. PLoS ONE.

