# Supplementary material for: Anterior Knee Pain Scale (AKPS): structural and criterion validity in Brazilian population with patellofemoral pain
Source: BMC Musculoskelet Disord. 2024 Jan 8;25:39. doi: 10.1186/s12891-024-07164-z (PMC10773022; doi:10.1186/s12891-024-07164-z)
Supplement: Supplementary file 1 — Additional file 1. The 11-item Anterior Knee Pain Scale (AKPS). Anterior Knee Pain Scale (AKPS) com 11 items. [file 12891_2024_7164_MOESM1_ESM.pdf]

The 11-item Anterior Knee Pain Scale (AKPS)

For each question, mark the latest choice (letter) which corresponds to your knee symptoms.

1. Limp
   a. None
   b. Slight or periodical
   c. Constant

2. Support
   a. Full support without pain
   b. Painful
   c. Weight bearing impossible

3. Walking
   a. Unlimited
   b. More than 2 km
   c. 1-2 km
   d. Unable

4. Stairs
   a. No difficulty
   b. Slight pain when descending
   c. Pain both when descending and ascending
   d. Unable

5. Squatting
   a. No difficulty
   b. Repeated squatting painful
   c. Painful each time
   d. Possible with partial weight beating
   e. Unable

6. Running
   a. No difficulty
   b. Pain after more than 2 km
   c. Slight pain from start
   d. Severe pain
   e. Unable

7. Jumping
   a. No difficulty
   b. Slight difficulty
   c. Constant pain
   d. Unable

8. Prolonged sitting with the knees flexed
   a. No difficulty
   b. Pain after exercise
   c. Constant pain
   d. Pain forces to extend knees temporarily
   e. Unable

9. Pain
   a None
   b. Slight and occasional
   c. Interferes with sleep
   d. Occasionally severe
   e. Constant and severe

10. Swelling
   a. None
   b. After severe exertion
   c. After daily activities
   d. Every evening
   e. Constant

13. Flexion deficiency
    a. None
    b. Slight
    c. Severe

Anterior Knee Pain Scale (AKPS) com 11 itens

Em cada questão, marque a letra que melhor descreve os atuais sintomas relacionados ao seu joelho.

1. Você caminha mancando?
   a. Não
   b. Levemente ou de vez em quando
   c. Constantemente

2. O seu joelho suporta o seu peso?
   a. Apóio totalmente, sem dor
   b. Apóio, mas sinto dor
   c. É impossível suportar o peso

3. Ao caminhar
   a. Não tenho limites para caminhar
   b. Caminho mais que 2 km
   c. Caminho entre 1 e 2 km
   d. Não consigo

4. Ao subir/descer escadas
   a. Não tenho dificuldade
   b. Sinto um pouco de dor ao descer
   c. Sinto dor ao descer e ao subir
   d. Não consigo

5. Ao agachar
   a. Não tenho dificuldade
   b. Sinto dor após agachamentos repetidos
   c. Sinto dor a cada agachamento
   d. Somente agacho com diminuição de meu peso (me apoiando)
   e. Não consigo

6. Ao correr
   a. Não tenho dificuldade
   b. Sinto dor após correr mais do que 2 km
   c. Sinto dor leve desde o começo
   d. Sinto dor intensa
   e. Não consigo

7. Ao pular/saltar
   a. Não tenho dificuldade
   b. Tenho um pouco de dificuldade
   c. Sinto dor constante
   d. Não consigo

8. Ao sentar com os joelhos flexionados/dobrados por período prolongado
   a. Não tenho dificuldade
   b. Sinto dor para me manter sentado após ter realizado exercícios
   c. Sinto dor constante
   d. A dor faz com que necessite estender (esticar) os joelhos de tempos em tempos
   e. Não consigo

9. Dor
   a. Nenhuma
   b. Leve e ocasional
   c. A dor atrapalha o sono
   d. De vez em quando é intensa
   e. Constante e intensa

10. Inchaço (edema)
   a. Nenhum
   b. Após esforço intenso
   c. Após atividades diárias
   d. Toda noite
   e. Constante

13. Sente dificuldade para flexionar/dobrar o joelho?
    a. Nenhuma
    b. Leve
    c. Muita
